# Assessing Autonomic Function from Electrodermal Activity and Heart Rate Variability During Cold-Pressor Test and Emotional Challenge

**DOI:** 10.1038/s41598-020-62225-2

**Published:** 2020-03-25

**Authors:** Shadi Ghiasi, Alberto Greco, Riccardo Barbieri, Enzo Pasquale Scilingo, Gaetano Valenza

**Affiliations:** 1 Department of Information Engineering & Bioengineering and Robotics Research Center E. Piaggio, School of Engineering, University of Pisa, Pisa, Italy; 2 Department of Electronics, Informatics and Bioengineering, Politecnico di Milano, Milano, Italy

**Keywords:** Biomedical engineering, Computer science

## Abstract

Standard functional assessment of autonomic nervous system (ANS) activity on cardiovascular control relies on spectral analysis of heart rate variability (HRV) series. However, difficulties in obtaining a reliable measure of sympathetic activity from HRV spectra limits the exploitation of sympatho-vagal metrics. On the other hand, measures of electrodermal activity (EDA) have been demonstrated to provide a reliable quantifier of sympathetic dynamics. In this study we propose novel indices of phasic autonomic regulation mechanisms by combining HRV and EDA correlates and thoroughly investigating their time-varying dynamics. HRV and EDA series were gathered from 26 healthy subjects during a cold-pressor test and emotional stimuli. Instantaneous linear and nonlinear (bispectral) estimates of vagal dynamics were obtained from HRV through inhomogeneous point-process models, and combined with a sensitive maker of sympathetic tone from EDA spectral power. A wavelet decomposition analysis was applied to estimate phasic components of the proposed sympatho-vagal indices. Results show significant statistical differences for the proposed indices between the cold-pressor elicitation and previous resting state. Furthermore, an accuracy of 73.08% was achieved for the automatic emotional valence recognition. The proposed nonlinear processing of phasic ANS markers brings novel insights on autonomic functioning that can be exploited in the field of affective computing and psychophysiology.

## Introduction

The sympathetic nervous system (SNS) plays a fundamental role in driving cardiovascular control dynamics together with the other branch of the Autonomic Nervous System (ANS), the parasympathetic nervous system^1,2^, and includes the regulation of blood pressure, respiration, and heart rate^1,3,4^. Many sympathetic neurons are part of the central nervous system (CNS) having origins in the thoracic and lumbar regions (T1-L2) in the spinal cord. Most of the sympathetic activity control on cardiovascular functioning is performed through noradrenergic neurons. In case of a drop in arterial pressure during situations such as anger, fear or excitement, the arterial baroreflex senses these changes in blood pressure through the baroreceptors, and the ANS acts to increase the vasoconstriction. As a result, the afferent input to the central autonomic nuclei is decreased, which in turn results in an increase in the sympathetic neural outflow. The resulting sympathetic influences in conjunction with the parasympathetic ones leads to an increase in heart rate, oxygen intake, and the blood supply to the heart and skeletal muscles. The release of epinephrine (adrenaline) and norepinephrine (noradrenaline), as well as the stimulation of sweat glands are also the results of a sympathetic nervous system elicitation^2,5^. The sympathetic vasodilator nerves in the human skin results from a cholinergic co-transmission, which releases acetylcholine as a primary neurotransmitter. The acetylcholine binds to muscarinic receptors on sweat glands to activate sweating, defining a sympathetic skin response^6^. An early quantification of the sympathetic activity has been performed through measurement of plasma or urinary norepinephrine. However, these indices may not be reliable due to their low sensitivity and non specific localization of the sympathetic response^1^. While a standard sympathetic activity measurement in humans refers to the invasive recording of the muscle sympathetic neural activity at the peroneal nerve^1,2^, non-invasive recordings of Heart Rate Variability (HRV) and Electrodermal activity (EDA) series show functional links with sympathetic and sympathovagal changes^1,7,8^.

One way to functionally assess the ANS branches is through the spectral analysis of HRV series, whose spectrum has been divided in two main bands of interest: the high frequency (HF) band, comprising oscillations between 0.15 Hz and 0.4 Hz, and the low frequency (LF) band, between 0.04–0.15 Hz. While HF power has been proven to be a reliable metric of vagal cardiac tone, as long as the respiratory frequency is within the HF band, the LF power is known to be influenced by both branches of the ANS^3–5^. Nevertheless, although with several limitations, the LF/HF ratio has been regarded as a measure of sympatho-vagal balance^3–5^. In the literature several attempts have been done to tackle the quantification issues carried by the spectral analysis of HRV^3,9,10^. Prior attempts employed other quantification methods on either HRV or ECG derived series to assess the prevalence of sympathetic or parasympathetic modulations. Extraction of symbolic indices obtained from short term heart-period variability^11^ and spectral analysis implementation on spontaneous beat-to-beat variability of RR and QT intervals^12^ are among them. On the other hand, EDA, linked to the activity of eccrine sweat glands controlled by the sudomotor nerves, has been proven to be a reliable measure of sympathetic activation. This is because the sympathetic nervous system is responsible for controlling the sudomotor activity^13,14^, and its assessment is based on EDA spectral analysis^7,15^.

Aiming at improving quantification of the sympatho-vagal balance, in this study we propose to combine EDA and HRV spectral estimates related to sympathetic and parasympathetic activity, respectively. ANS quantifiers from HRV and EDA signals have been exploited in recent studies in various application settings. Reviews of studies aiming at detecting emotional states and proposing automatic early stress recognition system through multimodal measurement of physiological signals (including HRV and EDA) are done in^16^ and^17^, respectively. Spectral quantifier of skin conductance, indexing sympathetic activity and high frequency spectral power of HRV, indexing parasympathetic activity have been shown to be useful in the evaluation of effort and/or stress tested during auditory tasks with different difficulty levels^18^. Moreover, authors in^19^ observed augmented sympathetic modulation in patients with schizophrenia by assessing the nonlinear coupling between HRV and skin conductance level.

However, the multimodal approaches used in the aforementioned studies have considered the spectral quantifiers of HRV and EDA time series as separate sources of information reflecting ANS changes. In fact, to the best of our knowledge no previous study aimed at investigating the effect of combining the spectral measures from the two time series in ANS dynamics. Moreover, since sympathetic-parasympathetic interplay can effectively be estimated through HRV bispectral analysis^20^, here we also investigate a proper combination of EDA and HRV bispectral metrics. In fact, the functional analysis of HRV in the frequency domain does not fully characterize the dynamical properties of the ANS activity on cardiovascular control, while HRV nonlinear analysis, especially referring to higher-order spectra, can do so^3,4,20,21^. Because of the many interactions and feedback mechanisms between sympathetic and vagal activities, cardiac dynamics is deemed to the output of a complex, nonlinear system^3,21^. Consequently, a dynamical vagal activation concurrent with a sympathetic one may lead to heart rate increase^21^.

In this study we exploit our recently proposed nonlinear point-process framework^20^, particularly referring to instantaneous spectral and bispectral HRV analysis. Briefly, this powerful statistical tool embeds the probabilistic generative mechanisms of heartbeat while providing model goodness-of-fit metrics^20^. The first-order moment of an inverse-Gaussian distribution characterizing a given heartbeat event is parametrized through a linear and nonlinear function of the past events, following a Wiener-Volterra representation with a Laguerre expansion of the kernels^20^.

Since the hereby proposed sympatho-vagal balance indices combining EDA metrics with HRV spectral and bispectral measures are estimated in a time-varying fashion, we also investigate the low frequency dynamics to capture slow trends and high frequency dynamics which captures the fast dynamical oscillations using a wavelet analysis, thus uncovering indices of dynamical information beyond first-order statistics (the standard average along the time would be equivalent to the study of indices of slow activity exclusively).

We validate the new proposed metrics on two experimental protocols: a standard stress test to evaluate cardiac autonomic function so-called cold-pressor test (CPT), and an emotional elicitation study using high-arousing video clips. Preliminary findings of this research were recently reported in^22,23^.

The CPT test was selected due to the efficient release of sympathetic neurotransmitters leading to marked vasoconstriction having an indirect effect in baroreflex mediating interactions^24–26^. Prior clinical and experimental studies have demonstrated significant cardiovascular responses to the cold stimuli,^27^. Specifically, the significant effect of CPT on left ventricular function has been demonstrated in a recent study^28^. These studies have shown major changes in heart rate and blood pressure during the CPT phase in healthy subjects^29^, as well as in patients with pathological conditions such as hypertension^30^, depression^31^, and anxiety^32^. Nevertheless, due to methodological limitations results were controversial^29–32^.

The emotional elicitation study has been chosen as a paradigmatic application exploiting reliable estimates of sympathovagal activity. Emotional processing, in fact, involves the activity of the so-called central autonomic network (CAN), comprising sympathovagal changes also driven by prefrontal cortex and amygdala functioning^33,34^. Accordingly, the study of emotional responses at the peripheral level has been widely investigated (see exemplary reviews in^35–38^. Nevertheless, reliable and specific ANS markers of emotional states in humans are still missing, possibly because of the non-satisfactory performance of state of the art about ANS metrics. Note that the quantifiers proposed in this study are intended to be included as functional set of ANS correlates of emotional states that can be retrieved noninvasively through wearable systems, in the frame of a nonlinear and non-stationary functional assessment. Methodological details, as well as experimental results and conclusions, follow below.

## Materials and Methods

### Subjects recruitment, experimental protocol, and acquisition set-up

**Figure 1:**
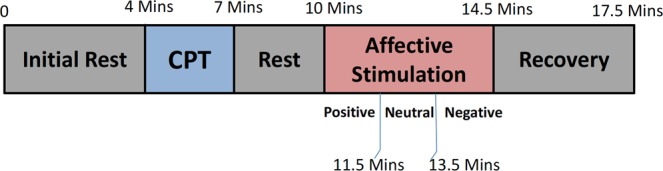
Timeline of the experimental protocol. The presentation order between CPT and affective elicitation, as well as the different kinds of emotional videos (positive, negative, and neutral) were randomized across subjects.

Twenty-six right-handed young healthy volunteers (8 females) from the University of Pisa participated in the study. Subjects did not have any history of neurological and cardiovascular diseases, or alcoholic or smoking habits. They were asked to avoid coffee and alcohol, as well as strenuous exercise, at least 2 hours before the laboratory visit^39–41^. Subjects aged between 21–32 (26 ± 3) with 60–100 kilograms of weight and 160–175 centimeters of height. All participants were screened using Patient Health Questionnaire (PHQ)^42^. This test intends to exclude the subjects with mental disorders for the experiment since the population under study is healthy control. Therefore, only participants with a PHQ score lower than 5 were included in the study. Valsalva maneuver and breathing holding was forbidden throughout the study. To ensure the hemodynamic stabilization, before the experiment participants were asked to sit in a comfortable chair, while watching a black screen. The experiment comprised two main phases: Cold-Pressor Test (CPT) and affective elicitation phase. The protocol timeline was as follows (see also Fig. 1):

- 4 minutes of resting state
- up to 3 minutes of CPT
- 3 minutes of resting state
- 4 minutes and half of emotional stimuli through videos
- 4 minutes of recovery.

The presentation order of CPT and emotional stimuli was randomized among subjects.

Concerning the CPT, participants were asked to submerge their left hand (i.e, the non-dominant hand) up to wrist into a tank filled of ice and water (crushed ice) with the temperature of 0–4 degrees centigrade for a period of 3 minutes^43^. The choice of 3 minutes is consistent with the average pain threshold of healthy subjects^44^. In case a subject could not tolerate this kind of elicitation, they were quickly moved to the next resting phase.

The emotional elicitation phase foresaw video-clips using a video-projector. The well-known Circumplex Model of Affect (CMA)^45^ was used for the emotional modeling through its valence and arousal dimensions. Valence refers to the pleasantness of the emotional perception, whereas arousal represents the strength of such a perception. In our study, the three categories of video-clips were associated with the following sessions: (1) high arousal and high positive valence, (2) neutral arousal/valence, (3) high arousal and high negative valence. The three categories have the same duration of 1 minute and 30 seconds and the presentation of the clips was randomized among subjects. The experiment ended by asking subjects for a complete self-evaluation of the stimuli through the visual analog scale questionnaire (VAS)^46^. Throughout the experiment, continuous electrocardiogram (ECG) and EDA signals were acquired using the BIOPAC MP35 device with a sampling rate of 500 Hz for further analyses^47^. The dedicated modules, ECG100C Electrocardiogram Amplifier and EDA100C Electrodermal Activity Amplifier from BIOPAC inc. were used to acquire the ECG signal and the electrodermal activity, respectively. The ECG100C ECG Amplifier has a noise voltage of 0.1 V (rms) in the bandwidth 0.05–35 Hz, a CMRR of 110 dB, a 16-bit ADC, and an amplitude resolution of 0.4 mV to record the D2 lead ECG signal. Pregelled Ag/AgCl electrodes for the ECG acquisition were placed following the Einthoven triangle configuration. EDA sensors were placed on the distal phalanx of the second and third finger of the dominant hand, imposing a DC voltage of 0.5V.

The RR series, defined as the interval between two successive QRS complexes, were derived by applying the automatic QRS complex detection algorithm on acquired ECG signals^48^. The technical artifacts due to the errors in R-peak detection algorithm were removed by applying cubic spline interpolation method^49^. The implementation was performed using Matlab software (R2017b version) and the Kubios HRV software^50^. The experimental procedure was approved by the “Comitato Etico Regionale per la Sperimentazione Clinica della Regione Toscana”, section “Area Vasta Nord Ovest” - Protocol n. 7803, Registry number 1072, approved on 18 Jan 2018. The recordings were carried out in agreement with the Declaration of Helsinki. Written informed consent was obtained from all subjects.

### Instantaneous estimates of vagal activity from inhomogeneous point-processes for heartbeat dynamics

Full details in this methodological framework are reported in^20^, referring to the recently proposed nonlinear parametric point-process model continuously characterizing the probabilistic properties of heartbeat events.

Briefly, assuming the history dependence, an inverse-Gaussian probability density function (*f*(*t*∣*H*_*t*_, *ξ*(*t*))), parametrized in its first-order moment (i.e., the instantaneous mean *μ*_*RR*_(*t*, *H*_*t*_, *ξ*(*t*))) and the shape parameter ${\xi }_{0}^{a}(t) > 0$, characterizes each heartbeat event as a function of the history *H*_*t*_ = (*u*_*j*_, *RR*_*j*_, *RR*_*j*−1_, . . . , *RR*_*j*−*M*+1_), with *t* the continuous time, *j* the index of previous R-wave from the ECG, and *RR*_*j*_ = *u*_*j*_ − *u*_*j*−1_ > 0 the time of successive R-wave events. The time varying parameters are in the vector $\xi (t)=[{\xi }_{0}^{a}(t),{g}_{0}(t),\{{g}_{1}(i,t)\},\{{g}_{2}(i,j,t)\}]$, which are estimated through a maximum log-likelihood procedure with Newton-Raphson algorithm^20^. Note also that this formulation is used to pre-process all heartbeat data to identify and eventually correct errors including peak mis-detection and ectopic beats^51^.

The first-order moment is defined as a nonlinear Volterra-Wiener autoregressive model:

1 $$\begin{array}{lll}{\mu }_{RR}(t,{H}_{t},\xi (t)) & = & {{\rm{RR}}}_{\widetilde{N}(t)}+{g}_{0}(t)+\mathop{\sum }\limits_{i=0}^{p}{g}_{1}(i,t)\ {l}_{i}(k)\\  &  & +\mathop{\sum }\limits_{i=0}^{q}\mathop{\sum }\limits_{j=0}^{q}{g}_{2}(i,j,t)\ {l}_{i}(k)\ {l}_{j}(k)\end{array}$$

where

2 $${l}_{i}(t)=\mathop{\sum }\limits_{n=1}^{\widetilde{N}(t)}{\phi }_{i}(n)({{\rm{RR}}}_{\widetilde{N}(t)-n}-{{\rm{RR}}}_{\widetilde{N}(t)-n-1})$$

is the output of the Laguerre filters before time *t* and

3 $${\phi }_{i}(n)={\alpha }^{\frac{n-i}{2}}{(1-\alpha )}^{\frac{1}{2}}{\sum }_{j=0}^{i}{(-1)}^{j}\left(\genfrac{}{}{0.0pt}{}{k}{j}\right)\left(\genfrac{}{}{0.0pt}{}{i}{j}\right){\alpha }^{i-j}{(1-\alpha )}^{j}$$

is the *i*^*th*^-order discrete time orthonormal Laguerre function, with (*n* ≥ 0) and *α* the discrete-time Laguerre parameter^20^. Kolmogorov-Smirnov (KS) test and associated KS statistics are employed to calculate the model goodness-of-fit^20^. The independence of the model-transfomed intervals are tested with the use of Auto-correlation plots^20^. Through this framework, three levels of quantitative tools defined in the time, spectral, and bispectral domains can be obtained.

Time domain estimates refer to the first-order (*μ*_RR_(*t*, *H*_*t*_, *ξ*(*t*))) and second-order (*σ*_*RR*_(*t*, *H*_*t*_, *ξ*(*t*))) moments of the underlying probability function *f*(*t*∣*H*_*t*_, *ξ*(*t*)) characterizing the heartbeat series at each moment in time^20^. Spectral quantifiers come from the linear power spectrum estimation *Q*(*f*, *t*, *H*_*t*_, *ξ*(*t*)), which depends on the linear terms *g*_1_(*i*, *t*) and related transformation from the Laguerre space to the Wiener-Volterra input-output terms^20^. Once estimated, the continuous *Q*(*f*, *t*, *H*_*t*_, *ξ*(*t*)) can be integrated within the low frequency (LF = 0.05–0.15 Hz) and high frequency (HF = 0.15–0.5 Hz) bands to derive instantaneous sympatho-vagal and vagal estimates, respectively. On the bispectral domain, the bispectrum ∣Bis(*f*_1_, *f*_2_, *t*)∣ is defined from the linear and nonlinear input-output Wiener-Volterra terms, and can be integrated in the LF and HF bands to derive the following quantifiers:

4 $$LL(t)={\int }_{{f}_{1}={0}^{+}}^{0.15}{\int }_{{f}_{2}={0}^{+}}^{0.15}Bis({f}_{1},{f}_{2},t)d{f}_{1}d{f}_{2}$$

5 $$LH(t)={\int }_{{f}_{1}={0}^{+}}^{0.15}{\int }_{{f}_{2}=0.1{5}^{+}}^{0.4}Bis({f}_{1},{f}_{2},t)d{f}_{1}d{f}_{2}$$

6 $$HH(t)={\int }_{{f}_{1}=0.1{5}^{+}}^{0.4}{\int }_{{f}_{2}=0.1{5}^{+}}^{0.4}Bis({f}_{1},{f}_{2},t)d{f}_{1}d{f}_{2}$$

Note that the bispectral quantifier *HH* has been demonstrated to provide estimates of vagal autonomic outflow^52^. All the introduced estimates are calculated with a 5 ms time resolution. More details about the formulation of the bispectrum can be found in^53^.

### Instantaneous estimates of sympathetic activity from EDA

The EDA signal reflects changes in the skin conductance induced by the sweat gland activity, which is directly controlled by the sympathetic branch of the ANS. Therefore, it is possible to rely on EDA signal processing to quantify the sympathetic dynamics through a proper functional analysis in the frequency domain^7,15^.

Specifically, a normalized EDA series, as a result of a Z-score transformation, is downsampled to 50 *Hz*. Afterwards, a time-frequency representation of the series is obtained through short-time Fourier transform and Welch periodogram, with a Blackman window of 60*s* and 59*s* overlap. Hence, a time-varying estimate of sympathetic outflow *EDA*_*Symp*(*k*)_ in *k* discrete time samples, within the interval (0, *T*), is obtained by integrating the time-frequency plane within the (0.045–0.25 Hz) band^7,15^.

Previously, a convex optimization modeling approach has been proposed for the decomposition of the skin conductance measure of EDA^54^. As the result of this decomposition, low and high frequency components of the skin conductance response (SCR) known as tonic and phasic activities, respectively, are separated. Mainly the SCR has been quantified by calculating the number of significant (above threshold) phasic driver peaks (*EDA*.*nSCR*), the sum of SCR amplitudes with respect to significant peaks (*EDA*.*SumAmpSCR*), the maximum value of phasic activity (*EDA*.*PhasicMax*). The quantification of the tonic component is done through the mean tonic activity computation (*EDA*.*Tonic*) as well.

### Proposed instantaneous indices of sympatho-vagal balance and related phasic dynamics

Aiming to derive reliable sympatho-vagal balance estimators, we combined the aforementioned *EDA*_*Symp*_(*k*), taken as a consistent measure of the sympathetic tone, and point-process derived measures from HRV linked to vagal activity, namely spectral power *HF* and bispectral powers *LL*, *LH*, and *HH*. Specifically, the following sympatho-vagal measures are defined:

7 $${S}_{HF}(k)=\frac{ED{A}_{Symp}(k)}{H{F}_{pp}(t){| }_{t=k}}$$

8 $${S}_{HH}(k)=\frac{ED{A}_{Symp}(k)}{H{H}_{pp}(t){| }_{t=k}}$$

9 $${S}_{LH}(k)=\frac{ED{A}_{Symp}(k)}{L{H}_{pp}(t){| }_{t=k}}$$

10 $${S}_{LL}(k)=\frac{ED{A}_{Symp}(k)}{L{L}_{pp}(t){| }_{t=k}}$$

**Figure 2:**
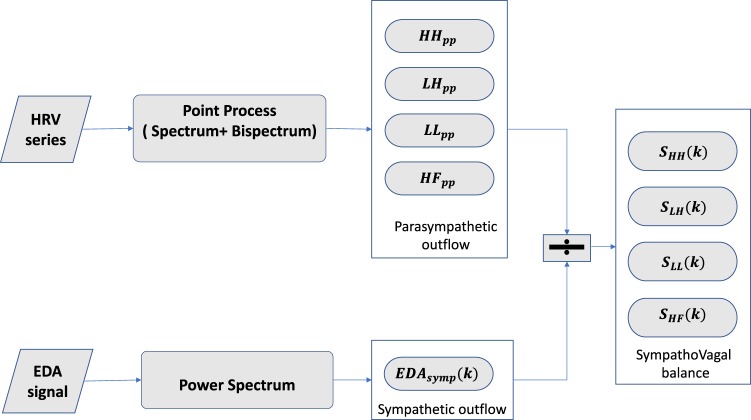
Block scheme on the derivation of the proposed indices of sympathovagal balance from EDA sympathetic markers and HRV-related parasympathetic markers.

**Figure 3:**
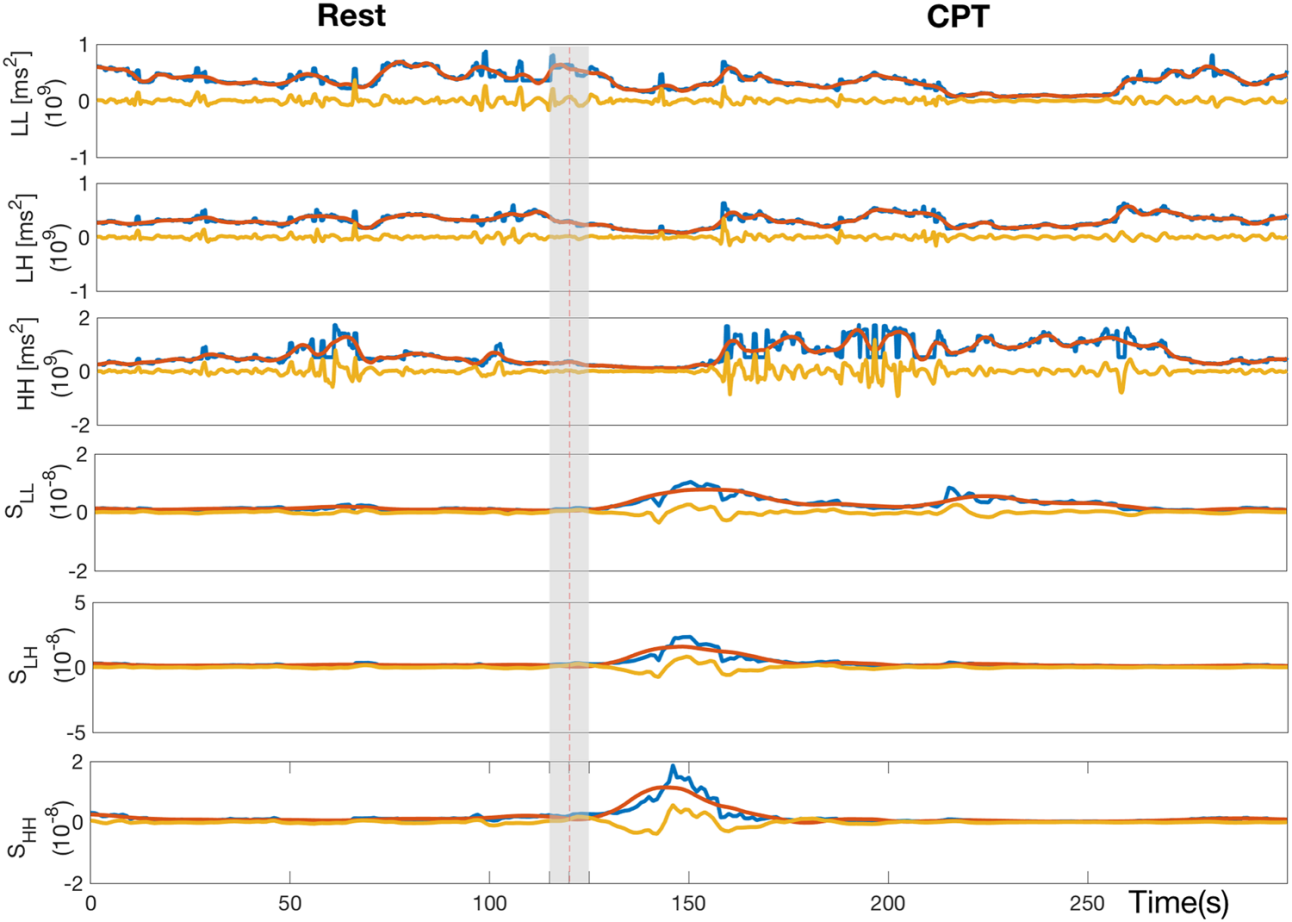
Exemplary decomposition of instantaneous bispectral estimates and their combination with *EDA*_*Symp*_ metrics in tonic and phasic dynamics. Data are from one exemplary subject during resting and CPT phases. Blue lines indicate the original series, whereas red and yellow lines represent the low and high frequency components, respectively. The 10s grey area centred at 120s indicates the transition between rest and CPT phases.

The derivation of sympatho-vagal measures is summarized in Fig. 2. We take a step further characterizing sympatho-vagal balance by (i) decomposing their time-varying dynamics into two components (1) the tonic activity representing a slow trend dynamic and (2) superimposed phasic component known to reflect high frequency oscillations of the time series and (ii) quantifying phasic dynamics using the median and area under the curve (AUC) metrics. Tonic and phasic decomposition of time-varying bispectral estimates *LL*, *LH*, and *HH* is further performed. An exemplary decomposition is shown in Fig. 3. Using a wavelet decomposition procedure^55^, tonic (S_ton_) and phasic (S_ph_) dynamics were derived from the first level of approximation coefficients and fifth level of detail coefficients, respectively, with a Daubechie5 function as the mother wavelet^55,56^.

### Classification for automatic valence recognition

Features were grouped into six categories. The first category *F*_1_ is from HRV measures, including instantaneous point-process estimates defined in the time and frequency domains and standard HRV time domain measures including Root Mean Square of the Successive Differences (RMSSD) and normalized mean number of times an hour in which the change in successive normal sinus (NN) intervals exceeds 50 ms (pNN50). *F*_1_ ∈  [*μ*_*RR*_, ${\sigma }_{RR}^{2}$, *RMSSD*, *pNN*50, *LF*_*pp*_, *HF*_*pp*_, *LF*_*pp*_/*HF*_*pp*_]. The HRV bispectral measures constitute the second feature category *F*_2_ ∈  [*LL*_*pp*_, *LH*_*pp*_, *HH*_*pp*_]. The third category *F*_3_ comprises EDA-related features estimated in the frequency domain *F*_3_ ∈  [*EDA*_*Symp*_, *S*_*HF*_]. Obtaining the decomposed tonic and phasic driver of SCR constitutes the fourth feature set: *F*_4_ ∈  [*EDA*.*nSCR*, *EDA*.*SumAmpSCR*,*EDA*.*PhasicMax*, *EDA*.*Tonic*]. The fifth category *F*_5_ comprises combined (tonic) indices from EDA and HRV bispectral features: *F*_5_ ∈  [*S*_*LL*_, *S*_*LH*_, *S*_*HH*_]. The sixth category *F*_6_ comprises median and AUC quantifiers of combined EDA and HRV bispctral phasic dynamics: ${F}_{{6}_{1}}\equiv$ Median(*S*_*phasic*_) and ${F}_{{6}_{2}}\equiv$ AUC(*S*_*phasic*_), where *S*_*phasic*_ ∈  [*LL*_*ph*_, *LH*_*ph*_, *HH*_*ph*_, ${{S}_{LL}}_{ph}$, ${{S}_{LH}}_{ph}$, ${{S}_{HH}}_{ph}$]. A list of all features used in this study along with the definitions and references from the literature are shown in Table 1.

**Table 1:** List of features used in this study with their definitions and references of their extraction.

Feature	Definition	Reference
***F***_1_		
*μ*_*RR*_[*ms*]	First-moment statistic of the Inverse Gaussian distribution in point process framework (Mean of RR series)	^67^
${\sigma }_{RR}^{2}$[*ms*^2^]	Second-moment statistic of the Inverse Gaussian distribution in point process framework (Variance of RR series)	^67^
*RMSSD*	Root Mean Square of the Successive Differences (RMSSD)	^50^
*PNN*50	Normalized mean number of times an hour in which the change in successive normal sinus (NN) intervals exceeds 50 ms	^50^
*LF*_*pp*_[*ms*^2^]	Integration of linear power spectrum estimate of RR series in the low frequency band (0.04–0.15 Hz)	^67^
*HF*_*pp*_[*ms*^2^]	Integration of linear power spectrum estimate of RR series in the high frequency band (0.14–0.45 Hz)	^67^
*LF*_*pp*_/*HF*_*pp*_	Ratio of LF and HF spectral powers	^67^
***F***_2_		
*LL*_*pp*_[*ms*^2^]	Double integration of the bispectrum of RR series in low and low frequency bands	^20^
*LH*_*pp*_[*ms*^2^]	Double integration of the bispectrum of RR series in low and high frequency bands	^20^
*HH*_*pp*_[*ms*^2^]	Double integration of the bispectrum of RR series in high and high frequency bands	^20^
***F***_3_		
*EDA*_*Symp*_	Integration of time-frequency plane of EDA signal within the 0.045–0.25 Hz band	^7^
*S*_*HF*_	Ratio between *EDA*_*Symp*_ and *HF*	This paper
***F***_4_		
*EDA*.*nSCR*	The number of significant (above threshold) phasic driver peaks	^54^
*EDA*.*SumAmpSCR*	The sum of SCR amplitudes with respect to significant peaks	^54^
*EDA*.*PhasicMax*	The maximum value of phasic activity	^54^
*EDA*.*Tonic*	Mean tonic activity computation	^54^
***F***_5_		
*S*_*LL*_	Ratio between *EDA*_*Symp*_ and *LL*	This paper
*S*_*LH*_	Ratio between *EDA*_*Symp*_ and *LH*	This paper
*S*_*HH*_	Ratio between *EDA*_*Symp*_ and *HH*	This paper
$${{\boldsymbol{F}}}_{{{\bf{6}}}_{{\bf{1}}}}$$		
*LL*_*ph*_	Median value of phasic decomposition of *LL*	This paper
*LH*_*ph*_	Median value of phasic decomposition of *LH*	This paper
*HH*_*ph*_	Median value of phasic decomposition of *HH*	This paper
${{S}_{LL}}_{ph}$	Median value of phasic decomposition of the ratio between *EDA*_*Symp*_ and *LL*	This paper
${{S}_{LH}}_{ph}$	Median value of phasic decomposition of the ratio between *EDA*_*Symp*_ and *LH*	This paper
${{S}_{HH}}_{ph}$	Median value of phasic decomposition of the ratio between *EDA*_*Symp*_ and *HH*	This paper
$${{\boldsymbol{F}}}_{{6}_{2}}$$		
*LL*_*ph*_(*AUC*)	Area under the curve of phasic decomposition of *LL*	This paper
*LH*_*ph*_(*AUC*)	Area under the curve of phasic decomposition of *LH*	This paper
*HH*_*ph*_(*AUC*)	Area under the curve of phasic decomposition of *HH*	This paper
${{S}_{LL}}_{ph}(AUC)$	Area under the curve of phasic decomposition of the ratio between *EDA*_*Symp*_ and *LL*	This paper
${{S}_{LH}}_{ph}(AUC)$	Area under the curve of phasic decomposition of the ratio between *EDA*_*Symp*_ and *LH*	This paper
${{S}_{HH}}_{ph}(AUC)$	Area under the curve of phasic decomposition of the ratio between *EDA*_*Symp*_ and *HH*	This paper

Time-varying dynamics were averaged within a 90s time window, which corresponds to the duration of either the neutral, pleasant and unpleasant elicitation. In order to minimize the inter-subject variability, for each subject the feature set related to a negative or positive stimulus was normalized by dividing for the value computed during a neutral session. These features were used to automatically classify emotional states associated with negative and positive elicitations through a standard nonlinear Support Vector Machine (SVM) with recursive feature elimination (RFE), which was validated through a Leave-One-Subject-Out (LOSO) procedure. RFE is a wrapper feature selection method that chooses a subset of features with size *r* among m features (*r*  <  *m*) that optimizes the performance of the SVM classifier^57,58^. The method is based on a backward sequential selection. Particularly, the feature that has the least impact on the SVM weight-vector norm is eliminated for each repetition. Therefore, the features are ranked and the SVM classification is repeated *m* times while the last ranked features are removed at each iteration. In this study, a recently developed nonlinear SVM-RFE which is an improved version of the previous RFE algorithm is implemented^59^. This developed method tackles the issue of the correlation bias of the previous method which makes it reliable if the input feature set contains highly correlated features.

The LOSO validation scheme foresees that, at each iteration, the SVM-RFE model is trained using feature samples from *N* − 1 subjects (where *N* is the total number of subjects), and tested on the feature samples from the left-out subject. This is iterated *N* times. Final feature ranking was obtained by summation of the ranks from each iteration.

**Figure 4:**
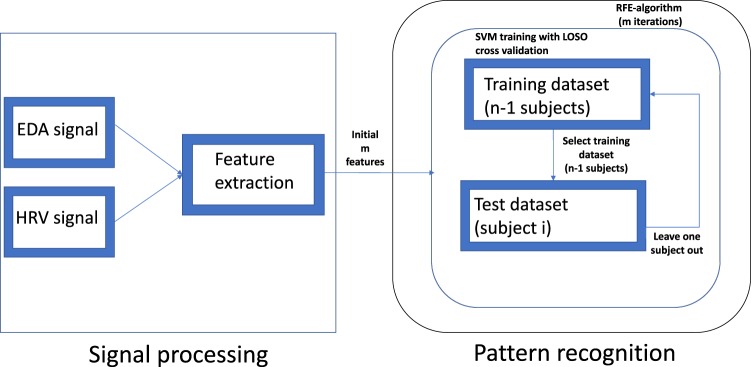
Processing chain of valence recognition.

Classification results are reported in terms of confusion matrix associated with the feature subset giving highest accuracy. Note that diagonal values of this matrix represent sensitivity and specificity of classification, which are defined as the proportion of actual positives and negative valence elicitations that were correctly identified, respectively. The recognition was performed using MATLAB v8.4 and an additional toolbox for classification^60^. The processing chain of the classification for valence recognition is depicted in Fig. 4.

## Experimental Results

We first report on a comprehensive characterization in the statistical sense of the proposed features performance by comparing resting vs. CPT phases. Given the non-Gaussianity of samples, as revealed by *p* < 0.05 from Kolmogorov-Smirnov tests with null hypothesis of data Normality, p-values for the Rest vs. CPT comparison are from Wilcoxon non-parametric tests for paired data, with null hypothesis of equal medians between samples. These results aim at ensuring that the phasic dynamics of the proposed linear and nonlinear sympatho-vagal measures carry significant information on ANS changes. Afterwards, we exploit this knowledge in recognizing positive vs. negative valence during the emotional video elicitations by applying the aforementioned SVM-RFE algorithm.

### Results on CPT

**Figure 5:**
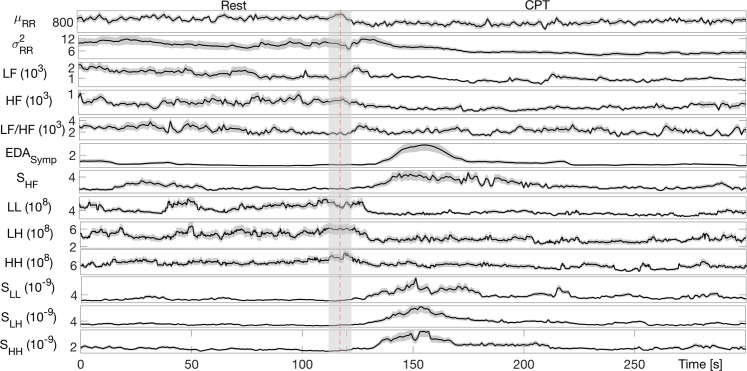
Dynamic tracking of instantaneous estimates of HRV and EDA averaged among twenty six subjects. From the top, the instantaneous *μ*_*RR*_, ${\sigma }_{RR}^{2}$, LF, HF, LF/HF, *EDA*_*Symp*_, bispectral measures (*HH*_*pp*_, *LH*_*pp*_, *LL*_*pp*_) and the proposed sympathovagal indices (*S*_*HH*_, *S*_*LH*_, *S*_*LL*_) are depicted. The median and MAD of each estimate are expressed through the continuous black line and the gray area. The red line at 120s separates resting and CPT phase by considering 10s transition between these phase indicated by the gray rectangle.

Group-wise dynamics for all features are shown in Fig. 5. Results from the Rest vs. CPT statistical comparison are shown in Table 2, where the feature time-varying evolution is expressed as Median ± $1.4826{\rm{MAD}}(X)/\sqrt{n}$, (where MAD(*X*) = Median(∣*X* − Median(*X*)∣), with *X* as the feature and *n* is the number of subjects in group) along the last 30*s* of rest and the first *t* s of CPT elicitation (where the *t* ∈ [30, 90, 180]).

**Table 2:** Median and MAD values of each feature among all 26 subjects considering the last 30s of rest and the last 30s, 90s, and 180s of CPT. the pvalues are as a result of pairwise statistical comparison obtained by Wilcoxon Test.

Feature	Rest(30s)	CPT(30s)	p-value	CPT(90s)	p-value	CPT(180s)	p-value
***F***_1_							
*μ*_*RR*_[*ms*]	868.69 ± 99.44	785.14 ± 96.63	**0.0009**	785.97 ± 128.23	**0.0004**	794.66 ± 122.07	**0.0002**
${\sigma }_{RR}^{2}$[*ms*^2^]	1028.56 ± 776.65	957.30 ± 576.27	0.50	659.79 ± 413.64	0.91	579.09 ± 362.41	0.81
*RMSSD*	0.04 ± 0.02	0.02 ± 0.005	**0.005**	0.03 ± 0.02	**0.004**	0.03 ± 0.01	**0.009**
*PNN*50	23.28 ± 15.10	16.27 ± 12.48	**0.03**	14.41 ± 11.32	**0.04**	13 ± 10.10	**0.01**
*LF*_*pp*_[*ms*^2^]	1038.49 ± 852.20	1165.42 ± 545.50	0.316	793.26 ± 401.85	0.14	984.45 ± 521.03	0.07
*HF*_*pp*_[*ms*^2^]	756.11 ± 485.39	486.56 ± 257.47	**0.05**	402.88 ± 223.02	0.08	449.28 ± 188.34	**0.03**
*LF*_*pp*_/*HF*_*pp*_	2.01 ± 1.38	2.03 ± 1.4	0.58	2.27 ± 1.53	0.98	2.48 ± 1.63	0.79
***F***_2_							
*LL*_*pp*_[*ms*^2^]	(6.52 ± 4.39). 10^8^	(2.81 ± 2.17). 10^8^	**0.001**	(3.06 ± 1.86)10^8^	**0.004**	(2.96 ± 1.44). 10^8^	**0.006**
*LH*_*pp*_[*ms*^2^]	(4.92 ± 3.02). 10^8^	(3.82 ± 2.65). 10^8^	**0.05**	(3.57 ± 2.09). 10^8^	0.06	(3.39 ± 1.86). 10^8^	**0.04**
*HH*_*pp*_[*ms*^2^]	(9.91 ± 7.49). 10^8^	(7.81 ± 6.34). 10^8^	0.25	(6.03 ± 5.08). 10^8^	0.19	(5.84 ± 4.03). 10^8^	0.25
***F***_3_							
*EDA*_*Symp*_	2.67 ± 2.28	0.98 ± 0.86	**0.003**	0.64 ± 0.51	**0.004**	0.65 ± 0.52	**0.006**
*S*_*HF*_	0.001 ± 0.001	0.003 ± 0.003	**0.003**	0.0026 ± 0.0021	**0.02**	0.002 ± 0.002	**0.02**
***F***_4_							
*EDA*.*nSCR*	0.88 ± 0.48	1.37 ± 0.85	**0.02**	1.49 ± 0.73	**0.04**	1.18 ± 0.53	0.36
*EDA*.*SumAmpSCR*	87.04 ± 65.20	134.37 ± 120.94	**0.003**	97.49 ± 85.55	**0.006**	88.44 ± 77.31	**0.03**
*EDA*.*PhasicMax*	61.38 ± 47.12	86.08 ± 75.47	**0.02**	54.38 ± 47.350	**0.02**	61.09 ± 51.67	**0.02**
*EDA*.*Tonic*	− 0.56 ± 0.88	− 0.89 ± 0.77	**0.007**	− 0.91 ± 0.68	**0.0002**	− 0.93 ± 0.73	**0.05**
***F***_5_							
*S*_*LL*_	(1.65 ± 1.32). 10^−9^	(6.56 ± 6.27). 10^−9^	**0.002**	(6.82 ± 5.63). 10^−^9	**0.0007**	(3.47 ± 3.20). 10^−^9	**0.0009**
*S*_*LH*_	(1.62 ± 1.16). 10^−9^	(5.98 ± 5.29). 10^−9^	**0.0012**	(5.40 ± 3.89). 10^−^9	**0.009**	(3.44 ± 2.34). 10^−9^	**0.05**
*S*_*HH*_	(8.55 ± 7.57). 10^−10^	(4.34 ± 3.65). 10^−9^	**0.001**	(3.04 ± 2.29). 10^−^9	**0.004**	(2.60 ± 1.89). 10^−9^	0.10
$${{\boldsymbol{F}}}_{{6}_{1}}$$							
*LL*_*ph*_	(6.72 ± 6.72). 10^9^	(6.53 ± 6.53). 10^9^	0.81	(2.25 ± 2.25). 10^10^	0.10	(3.51 ± 3.51). 10^10^	0.13
*LH*_*ph*_	(4.39 ± 4.39). 10^9^	(4.86 ± 4.86). 10^9^	0.24	(1.39 ± 1.39). 10^10^	0.53	(2.43 ± 2.43). 10^10^	0.79
*HH*_*ph*_	(7.22 ± 7.22). 10^9^	(4.86 ± 4.86). 10^9^	0.26	(1.96 ± 1.96). 10^10^	0.52	(3.72 ± 3.72). 10^10^	0.39
${{S}_{LL}}_{ph}$	(7.59 ± 7.51). 10^−9^	(2.41 ± 2.41). 10^−8^	0.87	(1.08 ± 1.08). 10^−7^	**0.04**	(1.84 ± 1.84). 10^−7^	**0.004**
${{S}_{LH}}_{ph}$	(6.85 ± 6.80). 10^−9^	(1.78 ± 1.78). 10^−8^	0.33	(1.04 ± 1.04). 10^−^8	0.10	(1.55 ± 0.55). 10^−^7	0.95
${{S}_{HH}}_{ph}$	(5.91 ± 5.88). 10^−9^	(1.33 ± 1.33). 10^−8^	0.14	(4.0009 ± 4.0009). 10^−8^	0.62	(6.71 ± 6.71). 10^−8^	0.16
$${{\boldsymbol{F}}}_{{6}_{2}}$$							
*LL*_*ph*_(*AUC*)	(6.72 ± 5.26). 10^9^	(6.54 ± 5.97). 10^9^	0.34	(2.25 ± 1.51). 10^10^	**0.013**	(3.51 ± 2.22). 10^10^	0.27
*LH*_*ph*_(*AUC*)	(4.38 ± 3.08). 10^9^	(4.86 ± 3.72). 10^9^	0.45	(1.39 ± 8.58). 10^9^	**0.05**	(2.43 ± 1.65). 10^10^	0.64
*HH*_*ph*_(*AUC*)	(7.23 ± 5.97). 10^9^	(4.87 ± 4.04). 10^9^	0.12	(1.96 ± 1.63). 10^10^	**0.04**	(3.72 ± 3.006). 10^10^	0.98
${{S}_{LL}}_{ph}(AUC)$	(7.59 ± 6.88). 10^−9^	(2.42 ± 2.34). 10^−8^	**0.005**	(10.81 ± 9.88). 10^−8^	**0.02**	(18.42 ± 15.53). 10^−8^	**0.003**
${{S}_{LH}}_{ph}(AUC)$	(6.84 ± 3.63). 10^−9^	(1.78 ± 1.69). 10^−8^	0.06	(10.43 ± 8.68). 10^−8^	**0.02**	(15.53 ± 11.02). 10^−8^	**0.002**
${{S}_{HH}}_{ph}(AUC)$	(5.91 ± 5.39). 10^−9^	(1.33 ± 1.29). 10^−8^	0.22	(4.01 ± 3.67). 10^−8^	**0.02**	(6.71 ± 5.87). 10^−8^	**0.001**

Among the *F*_1_ feature set (HRV time and frequency domain features), only *μ*_*RR*_ showed significant differences between rest and CPT for all the selected time windows. Significant differences were found also in *HF*_*pp*_ for the 30*s* and 180*s* windows. In the *F*_2_ feature set (HRV bispectral features), significant differences were found for *LL*_*pp*_ at all time windows, as well as for *LH*_*pp*_ taking estimates from 180*s* CPT. Remarkably, significant differences were found for the proposed features in *F*_3_ and *F*_5_, combining sympathetic EDA with linear and nonlinear vagal dynamics from HRV, but *S*_*LL*_ in the 180*s* time window. Concerning features from phasic dynamics in *F*_6_, significant p-values were found for ${{S}_{LL}}_{ph}$ for the 90*s* and 180*s* time windows. However, calculating the AUC of such phasic dynamics, bispectral features showed differences at 90*s*, whereas the ones from nonlinear sympatho-vagal balance were all different except for ${{S}_{HH}}_{ph}$ estimated at 30*s*.

### Valence recognition results

**Figure 6:**
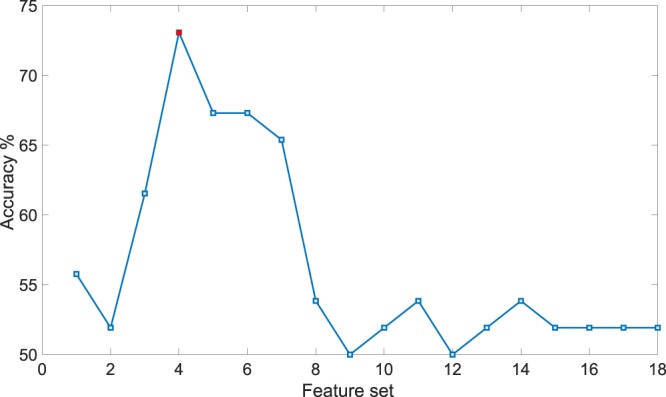
Valence recognition accuracy on validation set as a function of feature ranking selection implemented through the SVM-RFE-LOSO classifier.

The recognition accuracy as a function of the number of features, ranked through the SVM-RFE procedure, is shown in Fig. 6, whereas the confusion matrix associated with the best classification accuracy is reported in Table 3. This was achieved using four features, namely ${{S}_{LH}}_{ph}$, *LF*_*pp*_, *LH*_*ph*_, and ${{S}_{HH}}_{ph}$, with a balanced accuracy of 73.08% resulting in 75% and 71.43% of positive predictive value and negative predictive values, respectively.

**Table 3:** Confusion matrix for valence recognition using the final feature set. Values are expressed as percentages.

Feature set (Final)	Pleasant	Unpleasant
Pleasant	69.23%	30.77%
Unpleasant	23.08%	76.92%
Recognition accuracy: 73.08%		

Note that such most informative features come from spectral power in the LF, as well as from the proposed phasic dynamics of bispectral sympatho-vagal features. The complete feature set used for the classification, ranked through the SVM-RFE procedure, is reported in Table 4. As a comparison the classification results using other feature sets including only the indices extracted from HRV (*F*_1_ and *F*_2_) and the standard EDA indices (*F*_4_) are also reported (Tables 5 and 6, respectively).

**Table 4:** Ranked feature list used for valence classification.

Rank	Feature
1	${{S}_{LH}}_{ph}$*
2	*LF*_*pp*_[*ms*^2^]*
3	*S*_LH_*
4	*LH*_ph_*
5	${{S}_{HH}}_{ph}$
6	*HH*_*ph*_
7	LF/HF
8	*μ*_*RR*_[*ms*]
9	*LL*_*ph*_
10	*S*_*HH*_
11	*S*_*LL*_
12	*LL*_*pp*_[*ms*^2^]
13	*LH*_*pp*_[*ms*^2^]
14	${\sigma }_{RR}^{2}$[*ms*^2^]
15	*HH*_*pp*_[*ms*^2^]
16	*EDA*_*Symp*_
17	*HF*_*pp*_[*ms*^2^]
18	${{S}_{LL}}_{ph}$

**Table 5:** Confusion matrix for valence recognition using the feature set containing only HRV related indices.Values are expressed as percentages.

Feature set (*F*_1_,*F*_2_)	Pleasant	Unpleasant
Pleasant	65.02%	34.98%
Unpleasant	38.16%	61.84%
Recognition accuracy: 63.43%

**Table 6:** Confusion matrix for valence recognition using only standard measures of EDA. Values are expressed as percentages.

Feature set (*F*_4_)	Pleasant	Unpleasant
Pleasant	76.73%	23.27%
Unpleasant	39.77%	60.23%
Recognition accuracy: 68.48%		

## Discussion and Conclusion

In this study we investigated novel ANS metrics linked to sympatho-vagal dynamics by combining EDA and HRV spectral powers, as well as combining EDA spectral power with HRV bispectral power. To this end, while sympathetic estimates were gathered from the electrodermal activity (EDA), vagal estimates were from HRV series. Features from phasic activity series of HRV bispectral power and combined EDA spectra were investigated as well. Preliminary results of this study can be found in^22,23^.

More in detail, we combined features from EDA power spectra, namely *EDA*_*Symp*_, with instantaneous spectral and bispectral features from point-process models for heartbeat dynamics. The choice of a point process framework as a physiologically inspired probabilistic method lies in its uniqueness in parameterizing heartbeat dynamics continuously with any time resolution, without the need of an interpolation method. The quadratic autoregressive nonlinearities allows for the estimation of instantaneous bispectral measures and their decomposed high frequency component, which refers to the superimposed phasic activity. Time-varying information of such instantaneous phasic dynamics was quantified through central tendency (median) and AUC quantifiers. Previous studies accounted for first-and second-order moments exclusively to characterize instantaneous ANS activity^7,15,20,61–64^, therefore neglecting effective information from the superimposed phasic dynamics.

We first evaluated the proposed methodology during a paradigmatic ANS elicitation through prolonged cold-pressor test (CPT) in twenty six healthy volunteers. The significant decrease in *μ*_*RR*_ following the CPT onset is consistent with previous studies (see^29^ and references therein), and *RMSSD* and *pNN*50 also showed a significant decrease in specific time windows. Note that significant p-values were obtained from standard EDA measurements (*F*_4_) at all time intervals from the CPT onset, and a delayed response in *EDA*_*Symp*_ of about 30s from the CPT onset was also observed. We demonstrated that the sympatho-vagal balance can be better characterized by combining spectral and bispectral features derived from HRV with measures computed from EDA spectral analysis. The discriminative power of these measures is proven by the significant p-values associated with all time windows following the CPT onset, whereas the traditional LF/HF ratio was not able to discern CPT dynamics with respect to a previous resting state (Table 2 and Fig. 5).

Considering the 90s time windows from the CPT onset, while the *HH* did not show significant changes, the *HH*_*ph*_(*AUC*) was associated with a significant p-value. This result confirms the main hypothesis of this study for which relevant information on the ANS activity can be retrieved from the superimposed phasic behavior of HRV time-varying bispectral measures. Furthermore, the proposed new indices characterizing the cardiovascular control through EDA and HRV seem to provide a more effective indicator of the sympathovagal balance then traditional indices from HRV series only^7,15,29^.

The statistical comparison between the two valence levels showed no significant differences in the proposed feature sets. However, such features allowed to discern between pleasant and unpleasant affective elicitation through an automatic decision support system. Indeed, features associated with a non-significant p-value may provide meaningful information in a machine learning framework. By applying the SVM-RFE algorithm, the discriminant power of the high oscillations of nonlinear bispectral estimates are confirmed with respect to measures obtained from first-order moments only. A balanced accuracy of 73.08% is achieved for a valence recognition through a proper selection of both spectral and higher-order spectral features and their integration with power spectral estimates of EDA and related phasic activations (Table 4). As a comparison analysis, an HRV feature set including indices defined in the time and frequency domains (*F*_1_) and related bispectral estimates (*F*_2_) was fed to the classifier and an accuracy of 63.43% was obtained (Table 5), with 64% and 62.96% as positive predictive value and negative predictive value, respectively. On the other hand, a 68.48% accuracy with positive and negative predictive values of 65.52% and 71.43%, respectively, was associated with the use of a standard EDA feature set *F*_4_ (see Table 6). By comparing results in Tables 1 and 4, we may conclude that the proposed methodology could be a viable approach to integrate HRV and EDA dynamics in case of thermal and emotional elicitations tasks.

The different number of males and females in the dataset is a limitation of this study. Moreover, although the VAS phsychological test scores have proven the strong impact of stimuli in all subjects, there is no gold-standard procedure to evoke a sympathetic elicitation both at a cardiac and at a skin sympathetic outflow level. Furthermore, the emotional responses may not be consistent across the individuals, this increasing the inter-subject variability. However, it is important to remark that we did not aim to develop the most performant emotion recognition system, but rather we aimed to devise a computational method that effectively combines HRV and EDA information to asses autonomic changes during CPT and emotional processing. We performed the cold pressor test to induce changes in the autonomic control of cardiovascular dynamics different than an emotional elicitation task. Indeed, we could have performed other autonomic maneuvers including active and passive postural changes, lower body negative pressure, valsalva maneuver, handgrip test, and others. Moreover, the administration of the cold pressor test may have caused not negligible pain that might have affected the ANS estimates derived from HRV and EDA for further analyses. We are aware that specific lab conditions are required to study complex physiological phenomena related to emotional processing, and a direct translation of the proposed methodology to emotion recognition in ecological conditions may be challenging. Another limitation of this study is that in spite of the fact that the bispectral measure *HH* can be considered as a reliable index of cardiovascular vagal activity, the integration of *LH* and *LL* in the defined frequency bands may contain oscillations originating in the low frequency band which may not be linked to a a sympathetic activity exclusively.

Due to the asymmetric nature of the EDA signals, asymmetrically shaped Daubechies (dbN) wavelets were widely used to analyze the raw waveforms^65,66^. In our study, db5 (chosen among db1 to db10) was the most appropriate choice of the mother wavelet for a better decomposition of tonic and phasic activities. However, a procedure to select a specific mother wavelet should be carried out in the future. Moreover, although previous studies found a correlation between EDAsymp and sympathetic activity^7,15^, sympathetic sudomotor nerve activity may be different from the cardiac sympathetic dynamics. Further studies are therefore needed to quantitatively investigate the functional relationship between different sympathetic markers from physiological series.

Despite the mentioned limitations, this study opens new horizons for the study of autonomic control mechanisms and the functional assessment of emotional responses of human. Future work will be directed to the evaluation of the proposed methodology on data gathered during orthostatisc stressors such as head-up tilt, handgrip, and lower body negative pressure, as well as from different patient population with cardiovascular or mental disorders, or autonomic dysfunctions.

## Data Availability

The informed consent forms signed by the subjects prevent data from being publicly available. Data may be requested via email by researchers, upon reasonable request and verification of all ethical aspects, at: gaetano.valenza@unipi.it.
